# Exploring clinical outcomes in patients with idiopathic/inherited isolated generalized dystonia and stimulation of the subthalamic region

**DOI:** 10.1055/s-0043-1764416

**Published:** 2023-04-14

**Authors:** Clarice Listik, Jorge Dornellys Lapa, Sara Carvalho Barbosa Casagrande, Egberto Reis Barbosa, Ricardo Iglesio, Fabio Godinho, Kleber Paiva Duarte, Manoel Jacobsen Teixeira, Rubens Gisbert Cury

**Affiliations:** 1 Universidade de São Paulo, Center for Movement Disorders, Faculty of Medicine, Department of Neurology, São Paulo SP, Brazil.; 2 Universidade de São Paulo, Faculty of Medicine, Neurosurgery Division, Departament of de Neurology, São Paulo SP, Brazil.

**Keywords:** Deep Brain Stimulation, Dystonia, Cerebellum, Subthalamic Nucleus, Connectome, Estimulação Encefálica Profunda, Distonia, Cerebelo, Núcleo Subtalâmico, Conectoma

## Abstract

**Background** Deep Brain Stimulation (DBS) is an established treatment option for refractory dystonia, but the improvement among the patients is variable.

**Objective** To describe the outcomes of DBS of the subthalamic region (STN) in dystonic patients and to determine whether the volume of tissue activated (VTA) inside the STN or the structural connectivity between the area stimulated and different regions of the brain are associated with dystonia improvement.

**Methods** The response to DBS was measured by the Burke-Fahn-Marsden Dystonia Rating Scale (BFM) before and 7 months after surgery in patients with generalized isolated dystonia of inherited/idiopathic etiology. The sum of the two overlapping STN volumes from both hemispheres was correlated with the change in BFM scores to assess whether the area stimulated inside the STN affects the clinical outcome. Structural connectivity estimates between the VTA (of each patient) and different brain regions were computed using a normative connectome taken from healthy subjects.

**Results** Five patients were included. The baseline BFM motor and disability subscores were 78.30 ± 13.55 (62.00–98.00) and 20.60 ± 7.80 (13.00–32.00), respectively. Patients improved dystonic symptoms, though differently. No relationships were found between the VTA inside the STN and the BFM improvement after surgery ( *p* = 0.463). However, the connectivity between the VTA and the cerebellum structurally correlated with dystonia improvement ( *p* = 0.003).

**Conclusions** These data suggest that the volume of the stimulated STN does not explain the variance in outcomes in dystonia. Still, the connectivity pattern between the region stimulated and the cerebellum is linked to outcomes of patients.

## Resumo

**Antecedentes** A estimulação cerebral profunda (ECP) é um tratamento estabelecido para distonias refratárias. Porém, a melhora dos pacientes é variável.

**Objetivo** O objetivo do estudo foi descrever os desfechos da ECP da região do núcleo subtalâmico (NST) e determinar se o volume de tecido ativado (VTA) dentro do NST ou se a conectividade estrutural entre a área estimulada e diferentes regiões cerebrais estão associadas a melhora da distonia.

**Métodos** A resposta da ECP em pacientes com distonia generalizada isolada de etiologia hereditária/idiopática foi mensurada pela escala de Burke-Fahr-Marsden Dystonia Rating Scale (BFM) antes e 7 meses após a cirurgia. A soma dos volumes do NST nos dois hemisférios foi correlacionada com a melhora nos escores do BFM para avaliar se a área estimulada dentro do NST afeta o desfecho clínico. A conectividade estrutural estimada entre o VTA de cada paciente e as diferentes regiões cerebrais foram computadas usando um conectoma normativo retirado de indivíduos saudáveis.

**Resultados** Cinco pacientes com idade de 40,00 ± 7,30 anos foram incluídos. O BFM motor e de incapacidade basal eram de 78,30 ± 13,55 (62,00–98,00) e 20,60 ± 7,80 (13,00–32,00), respectivamente. Os pacientes melhoraram com a cirurgia, mas com variabilidade. Não houve relação entre o VTA dentro do NST e a melhora do BFM após a cirurgia ( *p* = 0.463). Entretanto, a conectividade estrutural entre o VTA e o cerebelo correlacionaram com a melhora da distonia ( *p* = 0.003).

**Conclusão** Os dados sugerem que o VTA dentro do NST não explica a variabilidade do desfecho clínico na distonia. Porém, o padrão de conectividade entre a região estimulada e o cerebelo foi relacionada com o desfecho dos pacientes.

## INTRODUCTION

Deep Brain Stimulation (DBS) is an already established treatment option for refractory dystonia. 1 2 Its main targets are currently the globus pallidus internus (GPi) and the subthalamic nucleus (STN) with similar motor and disability outcomes, ∼ 50 to 70% in isolated generalized/segmental inherited/idiopathic dystonia. 1 2 However, some patients still have poor outcome results (< 20% improvement in specific motor and disability scales like the Burke-Fahn-Marsden Dystonia Rating Scale [BFM]). 3

Globus pallidus internus DBS usually needs high stimulation energy, which results in shorter battery life. In addition, GPi stimulation can elicit stimulation-induced symptoms like freezing of gait and other fine motor and parkinsonian features. 4 In this scenario, the STN has emerged as an interesting target since lower stimulation energy is needed and the nucleus has several connections to different circuits in the brain. Nevertheless, the results also reveal wide variations in treatment outcome, 5 6 7 8 which highlights the need to determine why some patients improve after surgery and others do not, that is, which factors predict individual patient responsiveness.

Recent studies have revealed that the benefit of DBS may be based on the modulation of distant brain areas that are connected to the stimulation site. 9 10 This distant influence of DBS can be measured by studying the fiber tracts that structurally connect both the volume of the stimulated tissue and the corresponding distant area. It is hypothesized that the connectivity of the stimulation site to a specific brain network may be responsible for some of the DBS response. Here, we describe clinical outcomes of DBS that was applied to a few dystonic patients with idiopathic/inherited isolated generalized dystonia patients in an attempt to determine whether the electrode location and the connectivity profile between each patient correlates with dystonia improvement.

## METHODS

The present study was approved by our Institutional Ethics Review Board CAPPESQ (protocol number #48607515.5.0000.0068), and all patients gave written informed consent before being included in the study.

### Patients

Patients had generalized isolated dystonia of inherited/idiopathic etiology, 11 and all underwent DBS surgery due to refractory motor symptoms. Patients were excluded if they were < 18 years old, had other types of dystonia, or did not consent to participate.

### Study design

The present prospective study evaluated patients before and 7 months after surgery. First, patients were assessed before surgery. Then, patients were evaluated with the BFM scale, registering the motor (0–120) and disability subscale.

### Surgical technique

Before surgery, contrast-enhanced volumetric T1, T2, and susceptibility-weighted imaging (SWI) MRI scans were obtained in axial sections with a 1.5 T Siemens Espree scanner (Siemens, Munich, Germany). A stereotactic frame (Aimsystem, Micromar, Brazil) was placed on the head of the patient under local anesthesia, and a stereotactic contrast-enhanced computed tomography (CT) scan was performed.

Registration of image sets and target planning was subsequently performed using MNPS Planning Software (MEVIS neurosurgery planning system, MEVIS, Brazil). Based on diffusion-weighted imaging (DWI), tractography is a technique with great potential to characterize the in vivo anatomical position and integrity of white matter tracts. 12 It has proven its worth in neuroscience, neurology, and neurosurgery. 13 14 15 Furthermore, it is an invaluable tool in investigating structure-function relationships. Therefore, this technique was used to visualize the structural connectivity in the brain. 16

The STN target was chosen based on the atlas-based indirect targeting method and direct MRI-based targeting. 17 18 Coordinates related to the anterior commissure-posterior commissure line were used, and bilateral target points were located to the subthalamic nucleus: 2-3 mm posterior, 5-6 mm inferior, and 9-14 mm lateral to the midcommissural point. 19 The indirect target also includes the Schaltenbrand-Wahren atlas as a reference. An adjustment was performed to maintain the target in the posterior, dorsal, and lateral STN region related to motor function, with direct visualization of the nucleus on 3T MRI T2 and SWI sequences. 20

Quadripolar leads (model 6145 or 6149 [Abbot, Memphis, TN, USA] or model 3387 [Medtronic, Minneapolis, MN, USA]) were implanted in a way that the most ventral contact remained in the substantia nigra (SN). The determination of optimal stimulation parameters for each electrode was based on a detailed test-stimulation protocol implemented during the postoperative follow-up.

The accurate lead placement was confirmed with a postoperative CT scan registered to preoperative images using Lead-DBS software ( www.lead-dbs.org ).

### Lead location and volume of tissue activated (VTA)

Deep brain stimulation electrodes were localized in Lead-DBS (lead-dbs.org, Horn & Kühn. 2017). Postoperative tomography was coregistered to preoperative T1- and T2-weighted MRI using advanced normalization tools (stnava.github.io/ANTs/) 21 and then normalized to ICBM 2009b NLIN asymmetric (MNI) space 22 using the ANTs SyN method. 23 Deep brain stimulation electrodes were automatically reconstructed using the PaCER method 24 or the TRAC/CORE approach and manually refined. Once the electrode was localized, the VTA of the active contacts was estimated using a stimulation algorithm previously described by Baniasadi et al. 25

The VTA was based on patient-specific stimulation parameters recorded in the last hospital visit after surgery. The overlap between the VTA and the STN was calculated in mm ^3^ . The three parts of the STN were evaluated. The sum of the overlapping VTA/STN volumes from both hemispheres was correlated with the percent change in BFM (axial, appendicular, and total scores) to analyze whether the STN stimulated areas to influence clinical outcomes. Also, the overlap between the VTA and the SN was evaluated in the same manner.

### Connectivity assessment

Using VTAs as seed regions, structural connectivity estimates were analyzed using a normative structural connectome consisting of high-density normative fiber tracts based on 20 subjects. 26 Structural connectivity was calculated by extracting tracts passing through VTA and calculating the fiber count in a voxel-wise fashion in specific brain areas. 26 Brain parcellations were generally defined according to the automated anatomical labeling atlas 3, a probabilistic atlas covering several cortical and subcortical structural areas. 27 For the evaluation of connectivity to the insular cortex, the Hammers&Mith atlas 28 was used.

For motor correlation, we included regions of interest related to the pathophysiology of dystonia: precentral 29 30 and postcentral gyri, 29 cerebellum, 31 and substantia nigra. 32 33

### Statistical analysis

The Wilcoxon nonparametric test was used for the following BFM outcome score comparisons: baseline versus STN stimulation.

For the connectivity analysis, a linear regression was performed between the number of fibers connecting the VTA and the studied cortical or subcortical region and the improvement of clinical variables.

The independent variable was the BFM (axial, appendicular, and total scores) change (expressed in %). The dependent variable was the number of fibers connecting VTA and the studied region.

## RESULTS

Five patients completed the evaluation (4 males) aged 40.00 ± 7.30 years old with a baseline of BFM total motor and disability score of 78.30 ± 13.55 (62.00–98.00) and 20.60 ± 7.80 (13.00–32.00), respectively. Patients improved after surgery with a motor subscore of 68.10 ± 13.68 (53.50–87.00), *p* = 0.043, and a disability score of 15.00 ± 4.74 (9.00–21.00), *p* = 0.043. ( Table 1 ). There was variability in the clinical outcome after DBS ( Table 2 ).

**Table 1:** Burke-Fahn-Marsden dystonia scale results

		Baseline	STN	*p-value*
BFM	Eyes (0–8)	2.30 ± 0.97 (1.50–4.00)	1.90 ± 0.22 (1.50–2.00)	0.32
	Mouth (0–8)	5.40 ± 2.80 (2.00–8.00)	5.40 ± 2.80 (2.00–8.00)	1.00
	Speech and swallowing (0–16)	10.20 ± 3.90 (6.00–16.00)	7.80 ± 2.68 (6.00–12.00)	0.11
	Neck (0–8)	6.40 ± 1.70 (4.00–8.00)	4.40 ± 2.20 (2.00–6.00)	0.06
	Right arm (0–16)	12.00 ± 0.00 (12.00–12.00)	12.00 ± 0.00 (12.00–12.00)	1.00
	Left arm (0–16)	13.60 ± 2.20 (12.00–16.00)	13.40 ± 1.95 (12.00–16.00)	0.65
	Trunk (0–16)	11.20 ± 3.35 (8.00–16.00)	8.40 ± 3.58 (4.00–12.00)	0.06
	Right leg (0–16)	8.40 ± 3.58 (4.00–12.00)	7.40 ± 4.88 (1.00–12.00)	0.32
	Left leg (0–16)	8.80 ± 3.35 (4.00–12.00)	7.40 ± 4.88 (1.00–12.00)	0.32
	Total motor score (0–120)	78.30 ± 13.55 (62.00–98.00)	68.10 ± 13.68 (53.50–87.00)	0.043*
	Speech (0–4)	3.00 ± 0.70 (2.00–4.00)	2.60 ± 0.55 (2.00–3.00)	0.16
	Handwriting (0–4)	3.60 ± 0.55 (3.00–4.00)	2.60 ± 0.55 (2.00–3.00)	0.06
	Feeding (0–4)	2.80 ± 1.30 (1.00–4.00)	2.00 ± 1.22 (1.00–4.00)	0.10
	Eating/Swallowing (0–4)	2.80 ± 1.30 (1.00–4.00)	2.00 ± 1.87 (0.00–4.00)	0.10
	Hygiene (0–4)	2.20 ± 1.65 (1.00–4.00)	1.80 ± 1.30 (1.00–4.00)	0.32
	Dressing (0–4)	2.20 ± 1.65 (1.00–4.00)	1.60 ± 0.90 (1.00–3.00)	0.18
	Walking (0–5)	4.00 ± 2.35 (2.00–8.00)	2.60 ± 1.51 (1.00–4.00)	0.10
	Total disability score (0–29)	20.60 ± 7.80 (13.00–32.00)	15.00 ± 4.74 (9.00–21.00)	0.043*

Abbreviations: BFM, Burke-Fahn-Marsden dystonia scale; STN, subthalamic nucleus.

Data are presented as mean ± standard deviation (min–max), in which sample size is *n* = 5.

Notes: * *p* < 0.05 according to the Wilcoxon nonparametric test.

**Table 2:** Burke-Fahn-Marsden dystonia scale and clinical characterization of each patient. Sample size is *n* = 5

					Baseline BFM		Postoperative BFM		Improvement (%)	
	Age (years old)	Age of dystonia onset (years)	Genetic test available? (Y/N)/ type	Type of dystonia	Total motor score (0–120)	Total disability score (0–29)	Total motor score (0–120)	Total disability score (0–29)	Motor score	Disability score
Patient 1	44	10	Y/DYT- *THAP1* positive	Generalized with orolaryngeal isolated dystonia	75.5	18	53.5	15	29	16.5
Patient 2	50	20	Y/DYT- *THAP1* and DYT- *TOR1A* negative	Generalized with orolaryngeal isolated dystonia	62	15	59	9	5	40
Patient 3	39	6	Y/DYT- *PRKRA* positive	Generalized isolated dystonia	84	25	77	18	8.5	28
Patient 4	36	12	Y/DYT- *THAP1* and DYT- *TOR1A* negative	Generalized isolated dystonia	72	13	64	12	11	7.5
Patient 5	31	12	N	Generalized isolated dystonia	98	32	87	21	11	34

Abbreviations: BFM, Burke-Fahn Marsden Dystonia Rating Scale; N, no; Y, yes.

Notes: Patients with DYT-THAP1 and DYT-TOR1A negative were tested for both mutations, but tests for other mutation types were unavailable.

### Contact position and imaging analysis

**Figure 1:**
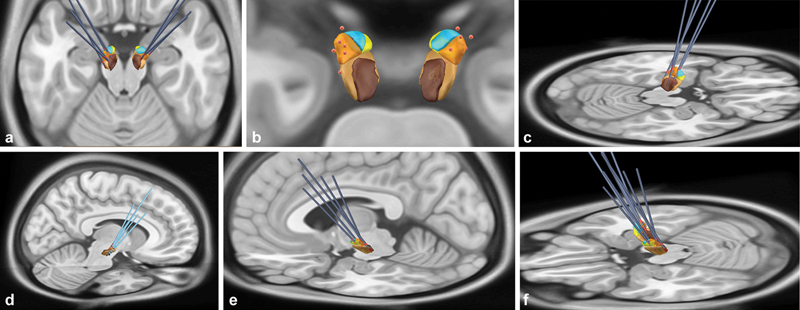
3D illustration of all electrodes and active electrode contacts. ( **A** ) All electrodes implanted in 5 dystonic patients, posterosuperior view. ( **B** ) Active contacts in STN, posterior view. C and D) Lateral view, right electrode. E and F) Lateral view, left electrode. Abbreviations: STN, subthalamic region; SN, Substantia nigra. Notes: Dark blue: electrodes; red: contacts; red point: active contacts; yellow: STN limbic subregion; light blue: STN associative subregion; orange: STN motor subregion; light brown: SN pars compacta; dark brown: SN pars reticulata.

There was no relation between the VTA intersection of the motor STN with an improvement of BFM after surgery ( *p* = 0.463) ( Figures 1 A and B ). Interestingly, the more anterior portions of the nucleus, as the associative STN ( *p* = 0.002) and limbic STN ( *p* = 0.0012), showed an association with improvement of BFM.

**Figure 2:**
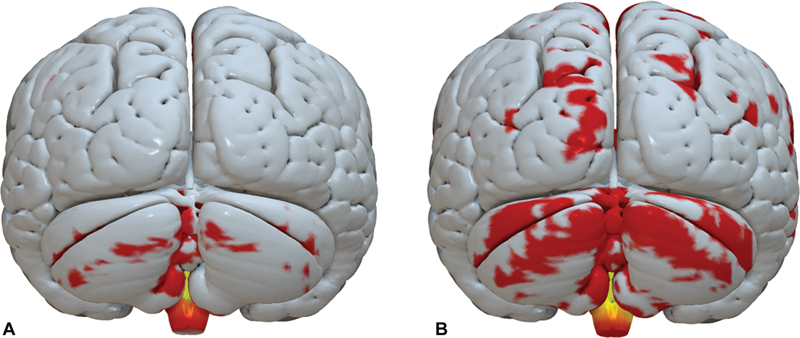
Illustration of structural connectivity. Topographies with higher connectivity (in red) to VTA in a responder (right) and a nonresponder (left). ( **A** ) Connectivity map of a nonresponder patient, posteroinferior view; ( **B** ) Connectivity map of a responder patient, posteroinferior view. Abbreviations: VTA, volume of tissue activated.

Evaluating the structural connectivity between the VTAs and cortical areas described above ( Table 3 ), we identified that the left precentral gyrus (r = - 0.64; *p* = 0.032) and the left postcentral gyrus (r = - 0.64; *p* = 0.028) correlated negatively with BFM improvement, although not statistically significant with the Bonferroni correction ( *p* < 0.008). Additionally, the left SN pars compacta (r = 0.68; *p* = 0.024) correlated positively with BFM improvement. Finally, there was a strong positive correlation between the DBS motor response with the cerebellum ( Figures 2 A and B ) that was statistically significant, the right lobule III (r = 0.75; *p* = 0.007) and vermis IX (r = 0.81; *p* = 0.003).

**Table 3:** Structural connectivity between the VTAs and other areas

		RHO*	*p-value*
Substantia nigra	Left pars compacta	0.68	0.024
Sensory and motor cortex	Left precentral gyrus	- 0.63	0.032
	Left poscentral gyrus	- 0.64	0.018
Cerebellum			
Central lobule-AL	Right III lobule	0.75	0.007
Lingula-AL	I/II Vermis	0.80	0.006
Uvula-AL	IX Vermis	0.81	0.003

Sample size is *n* = 5.

Note: * *p* < 0.008 with Bonferroni correction.

## DISCUSSION

Our primary conclusions are: i) the motor outcome after STN DBS in dystonia may differ between patients and the VTA inside the target (STN region) does not explain this variability in clinical outcomes; ii) the pattern of the connectivity between the region stimulated and specific cerebellar region may be responsible for the variance in outcome. These two points reinforce recent evidence that, although the targets for DBS in neurological disorders are normally determined by specific anatomical regions (nucleus or tracts), the ideal target may not necessarily be an anatomical structure in itself, but rather, a structurally connected area.

The classical DBS target in dystonia has been the GPi. Many recent studies with different types of dystonia have compared GPi and STN clinical outcomes showing similar results when motor and quality of life is concerned, with STN having a potential battery consumption advantage. 8 34 35 36 37 A study described a mean 6-month improvement in BFM movement score of 13.8 points, 35 which is in line with our results.

It is known that DBS outcomes in dystonia may vary because of several clinical and etiological factors. Our patients indeed had different motor outcomes after surgery. There are many reasons to explain this; for example, DYT- *TOR1A* responds better than DYT- *THAP1* , 7 younger patients and a shorter disease course are positive predictors, 5 phasic dystonia tends to respond better than a tonic one. 6

One may ask why it is important to study connectivity after DBS. It has been shown in Parkinson Disease (PD) that connectivity between the stimulation site and other areas (like the supplementary motor area and functional anticorrelation to the primary motor cortex) can predict clinical outcomes after surgery. 10 In Tourette syndrome, the connectivity between the thalamic centromedian-parafascicular region with the right frontal middle gyrus, the left frontal superior sulci region and the left cingulate sulci region structurally correlated with tic improvement. 9

In PD 38 and Meige Syndrome, 39 the VTA influences motor responses (presence of a sweet spot in STN). However, in Tourette syndrome, the VTA inside the target (centromedian nucleus-parafascicular region) did not correlate to motor improvement differences. 9

Interestingly, in our study, the motor STN did not correlate with an improvement in BFM after surgery. In contrast, the more anterior portions of the nucleus (the associative and limbic STN) did. While it is known that the dorsolateral part of STN is the sweet spot in PD, 38 our findings suggest that this may not be the case in dystonia.

A study with eight patients with focal and segmental dystonia and pallidal stimulation 40 found that the ventral GPi showed more robust measures of connectivity to the primary sensory cortex and posterior motor cortical regions. In contrast, dorsal GPi was more connected to motor and premotor regions. However, nothing is mentioned about the cerebellum. Another study with 15 patients with cervical dystonia and GPi-DBS found that the modulation of the primary motor putamen-posterior internal pallidum limb of the corticobasal ganglia loop predicted a successful DBS outcome. 41

There are also connectivity studies with dystonic patients without DBS. A resting-state functional MRI evaluated 13 patients with blepharospasm/Meige syndrome before and 4 weeks after botulinum toxin treatment. Patients had altered functional connectivity in the basal ganglia, cerebellar, primary/secondary sensorimotor, and visual areas. The toxin treatment modulated brain connectivity, including the cerebellum, and altered sensory input. 42 Untreated patients with cervical dystonia showed an imbalance of connectivity (both hyper- and hypo-) in the sensorimotor network and a disrupted somatosensory or sensorimotor integration. 43

The present study is the first to evaluate connectivity in dystonic patients submitted to STN DBS. Our results show that the motor outcomes of the patients strongly correlated with the cerebellum in the connectivity analysis. It has already been demonstrated in fMRI and transcranial magnetic stimulation (TMS) that there is an abnormal cerebellar activation and connectivity in dystonia compared to healthy volunteers. 44 45 Dystonia is a network disorder, and it is known that the cerebellum can modulate basal ganglia activity. 31 Therefore, perhaps the cerebellum could be an interesting target in dystonia for invasive and noninvasive stimulation.

Only one case report tried DBS targeting the bilateral superior cerebellar peduncle and dentate nucleus. The patient had a severe generalized fixed dystonia refractory to bilateral pallidotomy and intrathecal baclofen therapy, was bedridden, and was wheelchair-bound and able to move her arms and legs after the surgery. 46

The present study has some limitations, including the small sample size, which implied that further studies with larger samples would be interesting to replicate our findings. We tried to evaluate patients with a specific subtype of dystonia to diminish the heterogeneity of our sample. As seen above, most studies with dystonia and connectivity have a small sample size, even when they evaluate more common types of dystonia (that is, focal dystonia) with the more widely DBS target option for dystonia (that is, GPi). Also, our study does not have a control group, something that would be interesting to compare findings of different diseases. Other connectivity methods can be used to compare connectivity between healthy volunteers and dystonic patients, like TMS and fMRI. 47

Our findings show that the connectivity pattern could be another predictor factor in DBS outcome in dystonia. The volume of STN stimulated does not explain the different motor outcomes. The connectivity pattern in STN DBS for dystonic patients, particularly highlighting the cerebellum, influences treatment results and could be another predictor factor to consider.
